# Case report: A case of rectal metastatic squamous cell carcinoma from the esophagus

**DOI:** 10.3389/fonc.2023.1092868

**Published:** 2023-02-17

**Authors:** Junni Chen, Shuai Zhang, Jiawei Chen

**Affiliations:** Department of Radiation Oncology, Hainan General Hospital, Hainan Affiliated Hospital of Hainan Medical University, Haikou, Hainan, China

**Keywords:** rectal metastasis, squamous cell carcinoma, esophagus, treatment, case report

## Abstract

Esophageal cancer is prone to distant metastasis, and the prognosis is very poor; the occurrence of intestinal metastasis is extremely rare, with atypical clinical manifestations. We report a case of rectal metastasis after surgery for esophageal squamous cell carcinoma. The patient was a 63-year-old male who was admitted to the hospital due to progressive dysphagia. He was diagnosed with moderately differentiated esophageal squamous cell carcinoma after the surgery. He was not treated with chemoradiotherapy after surgery and had recurrent blood in his stool at 9 months post-surgery; rectal metastasis of esophageal squamous carcinoma was diagnosed based on postoperative pathology. Because the patient had a positive rectal margin, we used adjuvant chemoradiotherapy and carrelizumab immunotherapy, achieving very good short-term efficacy. The patient is currently in a state of tumor-free survival and is still being closely followed up and treated. Through this case report, we hope to deepen understanding of rare metastasis of esophageal squamous cell carcinoma and actively promote local radiotherapy plus chemotherapy and immunotherapy to improve survival.

## 1 Introduction

Esophageal cancer is a common malignant tumor of the digestive system. Its incidence ranks eighth in the world among malignant tumors, with mortality ranking sixth worldwide (1). The incidence of esophageal cancer is particularly high in China, mainly related to the following factors: nitrosamine compounds and mycotoxins; physical factors and geographical environmental factors; nutritional factors and lack of nutritional elements such as vitamins and trace elements such as zinc, selenium, and molybdenum; genetic factors; and eating habits, such as the long-term consumption of hot food, alcohol, spicy food, etc.

The pathological type of esophageal cancer in China is mainly squamous cell carcinoma, which is prone to distant metastasis and has very poor prognosis. The most common distant metastasis sites of esophageal cancer are the liver, distant lymph nodes, lungs, bone and brain. There are also a small number of literature reports on metastasis in other organs, including the skin, eyes, muscle, heart, skull, mandible, kidneys, breast, prostate, thyroid, spleen, bladder, small intestine, tonsils, and tongue (2). Intestinal metastasis of esophageal cancer is extremely rare, and the clinical manifestations are not typical. The specific symptoms mainly include intestinal perforation, bleeding and obstruction. Moreover, intestinal metastasis is difficult to detect at an early stage based solely on clinical manifestations and imaging examinations. Pathological examination and immunohistochemical examination are the gold standards for the diagnosis of this disease. The clinical data of a patient with rectal metastasis after surgery for esophageal cancer are reported herein to improve understanding of esophageal cancer metastasis at rare sites.

## 2 Case presentation

All procedures performed in human participants met the ethical standards of the institutional and/or national research committee(s) and with the Helsinki Declaration (as revised in 2013). Written informed consent was obtained from the patient.

**Figure 1:**
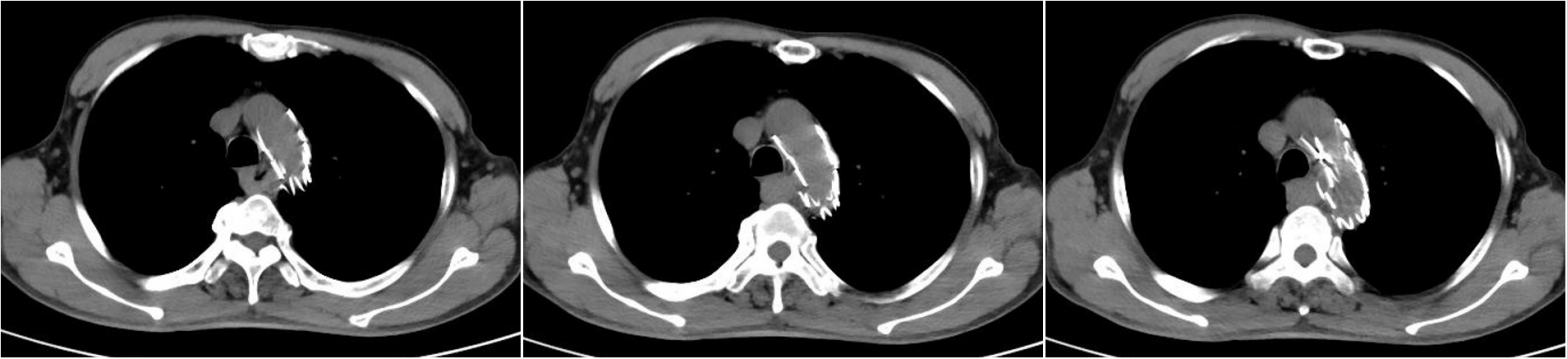
Chest CT scan on December 26, 2020, showing postoperative stent changes in the aortic arch and descending aorta and local thickening of the middle esophagus. (Because the patient showed allergy to contrast agents in the past, enhanced CT was not performed.).

**Figure 2:**
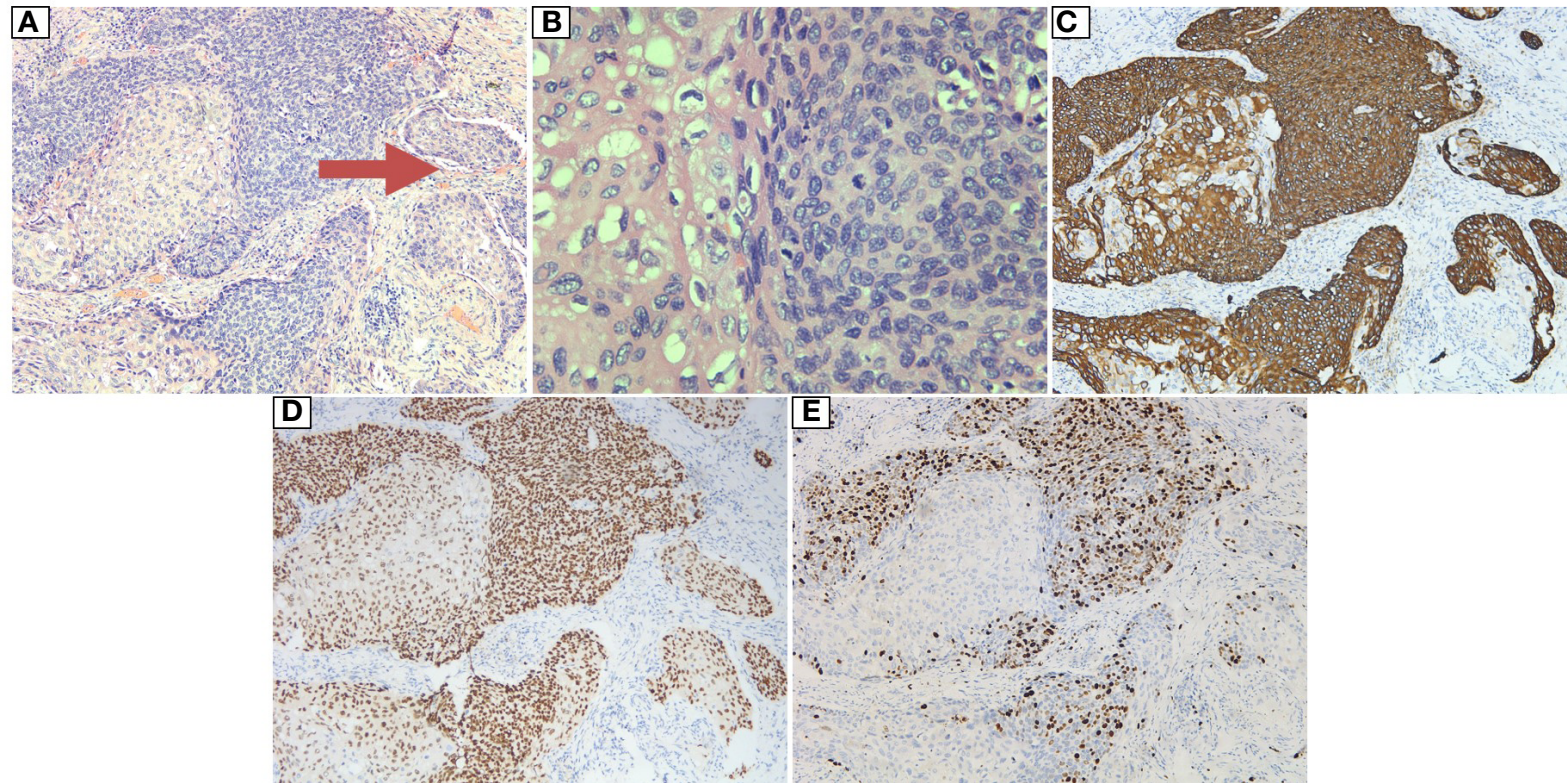
Postoperative pathology of the resected esophageal tumor: **(A)** HE: 10×10. The tumor cells were arranged in a solid nest and grew in an infiltrative manner. Intraventricular tumor emboli (indicated by arrow) were observed. **(B)** HE: 40×10. The tumor cells were polygonal in shape with an eosinophilic cytoplasm, and vacuoles were observed in some of the cytoplasm, with round, oval or irregular nuclei and rough nuclear chromatin; pathological mitosis was observed. **(C)** Magnification: 10×10. Immunohistochemical marker CK5/6 (+). **(D)** Magnification: 10×10. Immunohistochemical marker P40 (+). **(E)** Magnification: 10×10. Immunohistochemical marker KI67 (high proliferation index, +40%).

**Figure 3:**
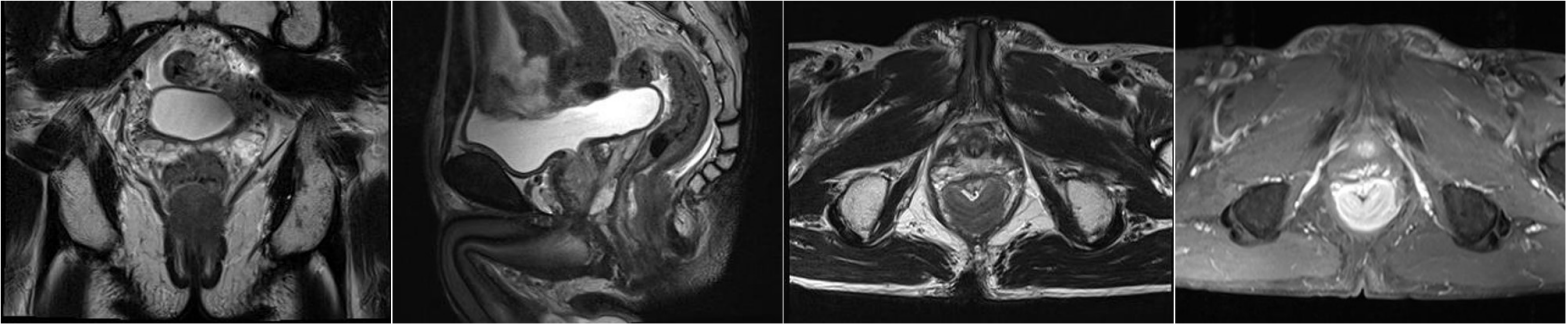
On September 30, 2021, pelvic MRI showed irregular thickening of the intestinal wall at the lower rectum (approximately 1.4 cm from the anal orifice), with the posterior wall predominating, accounting for approximately 2/3 of the circumferential wall, with a thicker area of approximately 1.8 cm. The enhanced scan showed obvious heterogeneous enhancement. The length of the lesion was approximately 3.8 cm, and multiple small lymph nodes were enhanced in the mesorectum.

**Figure 4:**
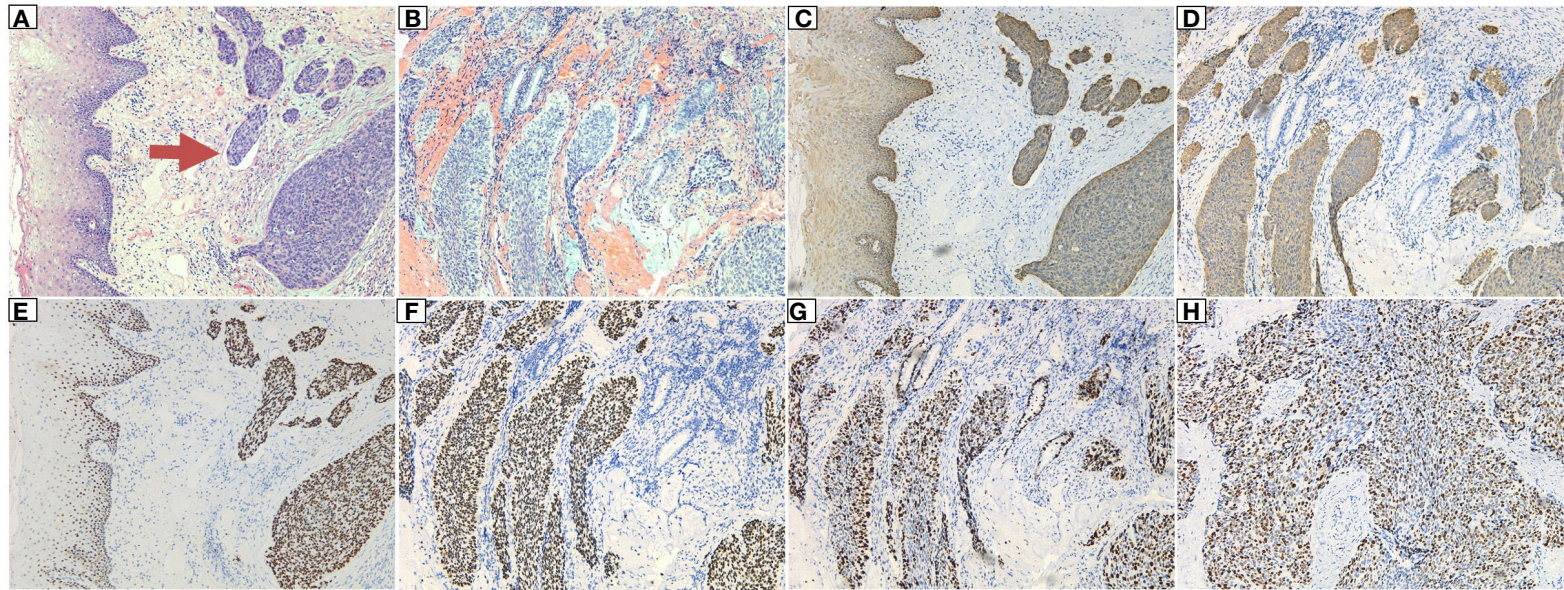
Postoperative pathology of the rectum: **(A)** The tumor cells had infiltrated the subepithelial interstitium in a nested, mass-like manner, and intravascular tumor emboli (indicated by the arrow) were visible, showing a clear boundary with the squamous epithelium near the rectum. **(B)** The tumor tissue showed mass and nested infiltrative growth, the epithelial-like cells outside the nest showed a basal cell-like arrangement, and residual mucinous glands were seen in the background of hemorrhage. **(C, D)** Magnification: 10×10. Immunohistochemical marker CK5/6 (+). **(E, F)** Immunohistochemical marker P63 (+). **(G, H)** Immunohistochemical marker KI67 (high proliferation index, +80%).

A 63-year-old male patient was admitted to the hospital on December 23, 2020, due to “progressive dysphagia” and underwent gastroscopic biopsy and chest computed tomography (CT) (**Figure 1**). The patient was diagnosed with moderately differentiated squamous cell carcinoma of the middle thoracic esophagus. Radical resection of the esophageal cancer was performed on December 28, 2020. Postoperative pathology indicated moderately differentiated esophageal squamous cell carcinoma, cancerous tissue infiltration into the outer membrane, no clear intraventricular tumor emboli or nerve invasion, no cancer at the cut edge, and cancer metastasis in lymph nodes (1/19). The lymph nodes were grouped as follows: left recurrent laryngeal nerve lymph node, 0/2; right recurrent laryngeal nerve lymph node, 1/2; subcarinal lymph node, 0/6; left gastric artery lymph node, 0/4; superior phrenic lymph node, 0/2; and middle paraesophageal lymph node, 0/3. Immunohistochemistry findings were as follows: P16 (-), P40 (+), and CK5/6 (+) (**Figure 2**). Postoperative radiotherapy and chemotherapy were not performed. In September 2019, the patient started having recurrent blood (bright red) in his stool 2-3 times/day. Colonoscopy showed a protruding lesion 3 cm from the anus (size, approximately 1.5 cm; surface, congested and hard; and surface mucosa, brittle), and biopsy was performed. Pathology indicated moderately differentiated squamous cell carcinoma (rectal). On September 30, 2021, pelvic magnetic resonance imaging (MRI) showed irregular thickening of the intestinal wall in the lower rectum and small lymph nodes in the mesorectum; neoplastic lesions were considered (**Figure 3**). Transanal resection of rectal tumors was performed on October 11, 2021. During the operation, a rectal mass approximately 3 cm × 4 cm in size was located. The texture of the mass was hard and slightly fixed. Postoperative pathological diagnosis was moderately differentiated squamous cell carcinoma (rectal tumor), and metastasis could not be excluded. Infiltration of the subadventitial adipose tissue, intravascular tumor emboli and nerve invasion were observed, with the tumor remaining at the radial resection margins. Immunohistochemistry results were as follows: CK (+), CK5/6 (+), P63 (+), Ki-67 (+, approximately 80%), CD34 (vascular +), CDX2 (-), CEA (-), CgA (-), D2-40 (lymphatic vessel +), P16 (-), P53 (-), and Syn (-) (**Figure 4**). Cell morphology and immunolabeling results confirmed that the esophagus was the origin. The patient was diagnosed with rectal metastasis of esophageal cancer. Due to the positive rectal margins, the TP regimen (paclitaxel 388 mg ivd Qd D1 + nedaplatin 112 mg ivd Qd D1 + camrelizumab 200 mg q3w) was initiated on November 19, 2021, for 1 cycle. Seven-field intensity-modulated pelvic radiotherapy was performed from November 23, 2021, to January 5, 2022. The radiation dose was as follows. In the first step, clinical target volume 1 (CTV1) of 45 Gy/25 F (including 2 cm above and below the tumor, the entire mesorectal area, the presacral area, the iliac lymphatic drainage area, and the obturator lymphatic drainage area) and CTV2 of 50 Gy/25 F (including the tumor and the mesorectal area 2 cm above and below the tumor) were administered. In the second step, the local dose of radiotherapy was increased by 10 Gy/5 F; that is, the dose in the tumor bed area reached 60 Gy/30 F. In addition, chemotherapy and immunotherapy were administered simultaneously. During radiotherapy, the TP regimen combined with carrelizumab immunotherapy was administered for 2 cycles. After completion of radiotherapy, 4 cycles of the TP chemotherapy regimen combined with immunotherapy were administered. The last date of chemotherapy was May 26, 2022. After systemic assessment, no local recurrence or distant metastasis was found. Carrelizumab (200 mg q3w) was continued for 2 cycles for maintenance therapy. The patient is still in the maintenance treatment period, which is scheduled to continue for 1 year.

## 3 Discussion

According to the latest report from the World Health Organization, there were 320,000 new cases of esophageal cancer in China in 2020, accounting for nearly half of cases worldwide, and 90% of the patients had esophageal squamous cell carcinoma (3). Esophageal cancer is prone to distant metastasis, and approximately 50% of patients are found to have distant metastasis at the time of diagnosis. The prognosis of patients with metastatic esophageal cancer is poor, with a 5-year survival rate of less than 5% (4). The English literature (PubMed) search for rare sites of esophageal cancer metastasis from 1982 to February 2017 by Osama Shaheen, excluded common sites of metastasis, i.e., the liver, lung, bone, adrenal gland, and brain, and retrieved 147 papers; 25 rare metastatic sites were identified, of which only 10 cases were reported to involve metastasis to the rectum (5). Jasbir Makker et al. reported a case of gastroesophageal junction adenocarcinoma with rectal metastasis in 2016 (6), and Ryohei Ono et al. reported a case of small bowel metastasis from lower thoracic esophageal cancer in 2018 (7). The overall prognosis of the patients in the above cases was extremely poor. Rectal metastasis of esophageal cancer is very rare, and the clinical manifestation is mainly blood in the stool. Immunohistochemistry plays an important role in determining the origin of the primary lesion of metastatic tumors. Important immunological markers for distinguishing squamous cell carcinoma from adenocarcinoma are P63 and CK5/6. In this case, adenocarcinoma was excluded based on CK5/6 (+) and P63 (+), and the diagnosis was squamous cell carcinoma. As the tumor was negative for CDX2 and CK20, an origin of the intestinal tract was basically excluded. In addition, the patient’s esophageal cancer was staged late. At approximately 10 months after esophageal cancer surgery, the patient was diagnosed with rectal squamous cell carcinoma after blood was found in the stool, and the interval between the two tumors was short. According to the principle of monism, the clinical, postoperative pathological and immunohistochemical results in this case were highly supportive of rectal metastasis of esophageal squamous carcinoma.

The mechanism of intestinal metastasis of esophageal cancer remains unclear. Esophageal cancer has the characteristics of extensive local growth, hematogenous dissemination, lymph node involvement, and implantation metastasis. Therefore, some studies suggest that metastasis can be achieved through the following pathways. 1. Implantation metastasis: In a patient with a history of middle esophageal squamous cell carcinoma, metastasis may be caused by implantation of cancer cells in the pelvic cavity during surgical incision of the diaphragm for esophagogastric anastomosis. In addition, the postoperative nutritional status of the patient may be poor and the immune barrier destroyed, prompting the rapid growth and invasion of cancer cells planted in the pelvic cavity (8). 2. Lymphatic metastasis: The esophagus has a rich lymphatic capillary network, which can intersect with abdominal collateral vessels and lymphatic vessels, and cancer cells can rapidly spread undetected in the abdominal cavity. Esophageal squamous cell carcinoma can spread extensively along the longitudinal lymphatic system of the esophagus and flow into a large number of widely distributed thoracic and abdominal lymph nodes based on the location of the tumor, thereby promoting metastasis (9). 3. Hematogenous dissemination: Hematogenous metastasis through the vertebral venous plexus is considered to be one of the possible mechanisms of metastasis to the abdomen (10). Furthermore, esophageal tumors are more susceptible to compression than other tumors, resulting in hematogenous or lymphatic dissemination, and integrins expressed on tumor cell exosomes can promote organ-specific metastasis (11). In the present case, we believe that the rectal metastasis was likely to have occurred through hematogenous dissemination. We suggest if abdominal pelvic implantation occurs in mid-stage esophageal cancer, it is often in the peritoneal fold above the peritoneal fold; in this case, the recurrent tumor was located in the rectal canal area 3 cm away from the anus, and there was no tumor recurrence above the peritoneal fold. If implant metastasis involves the anorectum, the outer membrane layer is first involved, and the postoperative pathology in this case suggests that the tumor had infiltrated into the subadventitial adipose tissue. Therefore, pelvic implant metastasis can be ruled out. We suggest that this case involved hematogenous dissemination.

National Comprehensive Cancer Network (NCCN) guidelines recommend a combination chemotherapy regimen based on cisplatin, docetaxel, and fluorouracil as first-line chemotherapy for metastatic esophageal cancer. Additionally, developments in molecular biology have led to promising new molecular targeted drugs for treatment of advanced esophageal cancer. For metastatic esophageal cancer, a stage III chemotherapy combined with immunotherapy trial (ESCORT-1st) was carried out in China. A study (12) confirmed that compared with chemotherapy, carrelizumab combined with chemotherapy can significantly prolong the median overall survival (mOS, 15.3 months vs. 12.0 months) of patients and reduce risk of death by 30%; it also significantly prolonged median progression-free survival (mPFS, 6.9 months vs. 5.6 months) and reduced the risk of disease progression by 44%. The carrelizumab combined chemotherapy group also had a higher objective remission rate (ORR, 72.1% vs. 62.1%) and a longer duration of remission (DoR, 7.0 months vs. 4.6 months). Therefore, “carrelizumab + cisplatin + paclitaxel” is recommended as the first-line treatment regimen for metastatic esophageal cancer in the new edition of the “Chinese Society of Clinical Oncology (CSCO) Guidelines for the Diagnosis and Treatment of Esophageal Cancer.” Because our patient had rectal metastasis of esophageal squamous cell carcinoma with a positive rectal margin, we combined pelvic intensity-modulated radiotherapy on the basis of the first-line treatment plan to reduce distant metastasis and limit recurrence of local metastases. The patient successfully completed radiotherapy and chemotherapy and is currently undergoing maintenance therapy with carrelizumab. Very good short-term efficacy was achieved. Currently, the patient is in a state of tumor-free survival without any discomfort and is still under close follow-up and treatment.

## 4 Conclusions

In summary, rectal metastasis of esophageal cancer is relatively rare, with strong invasiveness, poor prognosis, and short survival. Therefore, early detection and diagnosis can improve the prognosis of patients to a certain extent. Through this case study, we deepen understanding of the rare metastasis of esophageal squamous cell carcinoma and actively promote local radiotherapy plus chemotherapy and immunotherapy to generate better survival benefits.

## Data availability statement

The original contributions presented in the study are included in the article/Supplementary Material. Further inquiries can be directed to the corresponding author.

## Ethics statement

Written informed consent was obtained from the individual(s) for the publication of any potentially identifiable images or data included in this article.

## Author contributions

JuC collected, sorted, and analyzed the data and drafted the manuscript. SZ collected and sorted the data. JiC reviewed and revised the manuscript. All authors contributed to the article and approved the submitted version.
